# Differential Induction of Endoplasmic Reticulum Stress Signaling by Antibody Isotypes: Implications for Plasma Cell Differentiation

**DOI:** 10.1002/eji.202451428

**Published:** 2025-04-25

**Authors:** Kunie Obayashi, Tomomitsu Doi, Kazuhiro Sumida, Motoyoshi Endo

**Affiliations:** ^1^ Department of Molecular Biology University of Occupational and Environmental Health Kitakyushu Japan

**Keywords:** BiP, ER stress, IgE, plasma cell differentiation, protein structure

## Abstract

IgE induces stronger ER stress than IgG1 due to its constant region, particularly the Cε3 domain, which binds BiP more efficiently. Genetic and structural analyses confirmed IgE's higher BiP‐binding capacity. ER stress, driven by IRE1‐XBP1 signaling, regulates plasma cell differentiation, suggesting IgE‐specific mechanisms in immune responses.

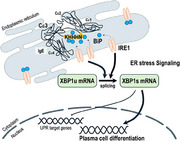

**Abbreviations**
BiP: binding immunoglobulin protein
ER: endoplasmic reticulum
Ig: immunoglobulin
IRE1: Inositol‐Requiring Enzyme 1
XBP1: X‐box binding protein 1

Although class switching to both IgG1 and IgE is induced by the Th2 cytokines, interleukin 4 (IL‐4) and IL‐13, the blood concentration of IgE is much lower compared with that of IgG1. We previously demonstrated that IgE class switching is not as limited as expected in vitro with adequate cytokines and nutrients; however, dense culture conditions hinder IgE, but not IgG1, protein synthesis and transport from the endoplasmic reticulum to the cell surface [1, 2].

IgE+ B cells are more prone to terminal differentiation into short‐lived plasma cells compared with IgG1+ B cells [3]. Plasma cells require the expansion of the endoplasmic reticulum (ER) to produce an enormous amount of immunoglobulin, and their differentiation requires ER stress signaling, particularly X‐box binding protein 1 (XBP1) [4]. XBP1 is activated by splicing of its mRNA by IRE1 [5]. IRE1 is repressed by the ER chaperone Binding immunoglobulin protein (BiP), while unfolded proteins relieve IRE1 inhibition by sequestering BiP [6].

The differences in the ability of each isotype to induce ER stress signaling have not been examined. In this study, we evaluated the ability of IgG1 and IgE to induce ER stress signaling using genetic engineering and structural simulations. We examined the induction of ER stress signaling in IgG1 or IgE+ B cells by measuring *Xbp1* mRNA splicing. The ratios of spliced to unspliced *Xbp1* mRNA in IgE+ B cells were much higher compared with those in IgG1+ B cells (Figure 1A). Next, we examined which ER stress signaling pathway was involved in plasma cell differentiation. The PKR‐like kinase (PERK) and IRE1‐XBP1 are major signaling pathways that transduce ER stress signals to the nucleus to induce the ER stress response. We observed that the relative level of the plasma cell markers CD138+ TACI+ cells and CD138+ CD98+ cells decreased with the IRE1 endonuclease inhibitor STF‐083010, but not with the PERK inhibitor GSK‐2656157, even at a concentration that suppressed PERK‐dependent *CHOP* mRNA induction (Figure 1B; Figures S1–S3). Therefore, plasma cell differentiation is dependent on IRE1‐XBP1 signaling [4].

**FIGURE 1:**
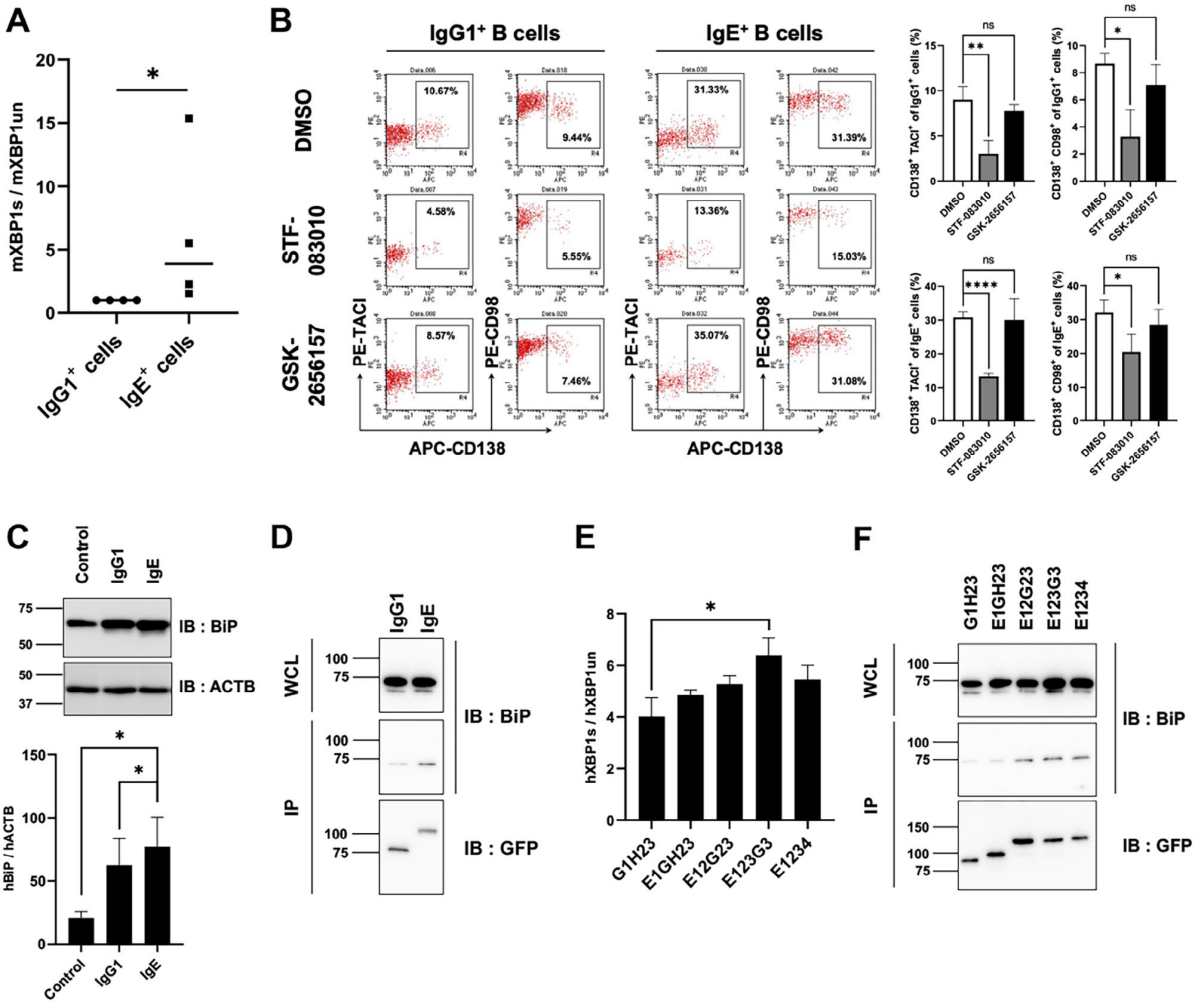
The Cε3 domain is responsible for the increased ability to induce ER stress signaling in IgE. (A) IgG1+ and IgE+ cells were sorted from splenic B cells cultured in vitro. The relative ratios of the spliced *Xbp1* transcript to the unspliced transcript are shown. (B) Splenocytes were cultured for 4 days with anti‐CD40 antibody and IL‐4 in the presence or absence of STF‐083010 or GSK‐2656157. The percentage of CD138+ TACI+ or CD98+ CD138+ cells is shown. (C) Plasmids expressing GFP (control), CD8α‐IgG1‐GFP (IgG1), and CD8α‐IgE‐GFP (IgE) were introduced into 293T cells. After 24 h, hBiP protein was detected by western blot analysis. hBiP protein expression levels normalized to actin. (D) CD8α‐IgG1‐GFP or CD8α‐IgE‐GFP was introduced into 293T cells. CD8α‐Ig‐GFP was immunoprecipitated, and BiP protein and GFP were detected by western blot analysis. (E) Relative ratios of spliced to unspliced *Xbp1* transcripts in 293T cells are shown. (F) BiP binding of chimeric proteins was examined by co‐immunoprecipitation as in (D). Data represent the means ± SD (*n* = 3), ns, no significance, *p < 0.05, **p < 0.01, ****p < 0.0001 (Student *t*‐test, or Mann–Whitney *U*‐test).

Next, we examined the ability of the IgG1 and IgE constant regions to induce ER stress signaling by introducing chimeric proteins containing the constant regions of IgG1 or IgE with the extracellular domain of CD8α, instead of the variable region, into 293T cells. We analyzed BiP protein expression to estimate ER stress signaling because ER stress induces its expression [7, 8]. Although the IgG1 and IgE chimeras induced BiP protein expression, its expression in the IgE chimera‐expressing cells was higher compared with that in the IgG1‐expressing cells (Figure 1C; Figures S4, S8). Moreover, we analyzed BiP binding to the IgG1 and IgE constant regions by immunoprecipitation. The amount of BiP protein precipitated with the IgE constant region was higher compared with that of the IgG1 constant region (Figure 1D; Figure S9). The results indicate that the IgE constant region is more prone to bind BiP and induce ER stress signaling compared with the IgG1 constant region.

The IgE constant region consists of four domains. To determine which domain is responsible for inducing ER stress in the IgE constant region, we constructed chimeric genes consisting of domains and hinge region from the IgG1 and IgE constant regions and introduced them into 293T cells (Figure S5). The construct in which the C‐terminal side was replaced by the IgE constant region from the third region enhanced ER stress and increased binding to BiP (Figure 1E,F; Figure S10). Unexpectedly, the ER stress signal induced by E1234 was weaker compared with that induced by E123G3 (Figure 1E,F; Figure S10). This likely occurred because the cognate Cε4 domain stabilizes the structure of the Cε3 domain. Overall, the results indicate that the Cε3 domain is responsible for inducing ER stress in the IgE constant region through BiP binding.

ER stress signaling is induced by the binding of the ER chaperone BiP to the unfolded region of nascent proteins. The region prone to denaturation and BiP binding in the Ig constant regions was predicted by computer‐assisted simulations. The hydrophobicity of the amino acids (Kyte–Doolittle index) [9] of the third regions of IgG1 and IgE (IgG1‐Cγ2, and IgE‐Cε3) was plotted (Figure 2A). Although the hydrophobic and hydrophilic residues generally alternate in stable structures, two regions that stretch the hydrophilic residues are present in the IgE‐Cε3 region. The BiP‐binding site in the third region was predicted from the primary amino acid sequence using the ChaperISM algorithm [10] (Figure 2A,B). One of the predicted BiP‐binding sites corresponds to the second hydrophilic region in the IgE‐Cε3 region, but not in the IgG1‐Cγ2 region. This region protrudes outside of the protein structure of the IgE constant region as predicted by AlphaFold2 (https://alphafold.ebi.ac.uk), and it is presumed that it may readily bind to BiP (Figure 2C). To validate the BiP‐binding motif of IgE predicted by computer modeling, we constructed chimeric proteins containing only the IgE (KHHHN) motif and the counterpart sequence from IgG1 (EEQFN) and examined their BiP‐binding ability in 293T cells (Figure S6). Although a single KHHHN motif was not enough to induce ER stress signaling (Figure S7), the BiP‐binding ability of the KHHHN motif was higher compared with that of the EEQFN sequence (Figure 2D; Figure S11).

**FIGURE 2:**
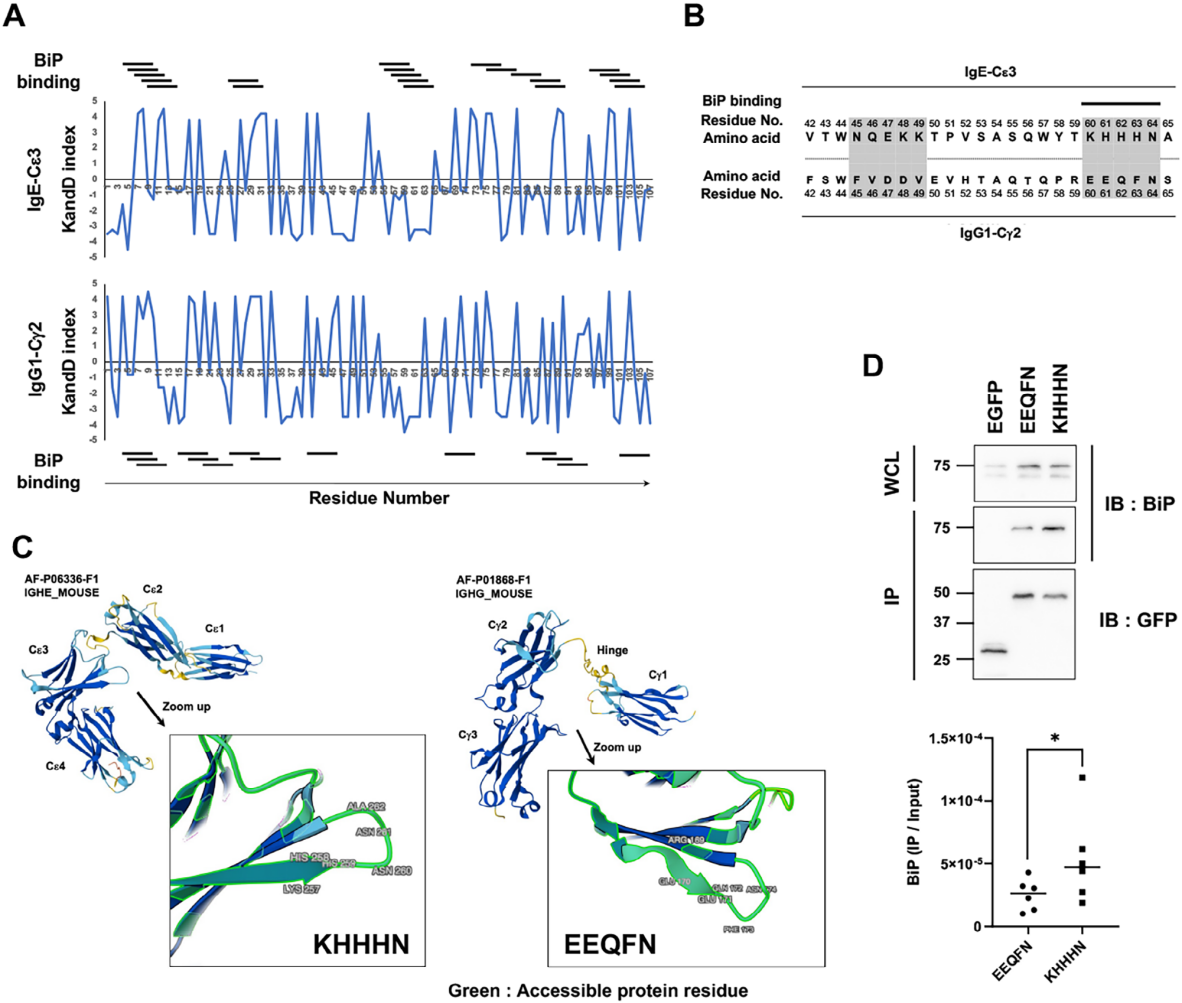
Prediction of the BiP‐binding region in the IgE‐Cε3. (A) Kyte–Doolittle (K and D) indexes of amino acids in the third regions of IgG1 and IgE are plotted. The horizontal lines above and below each graph indicate the BiP‐binding sequences predicted from the primary sequences using the ChaperISM algorithm. (B) Amino acids 42–65 of IgE and IgG1 are shown. The hydrophilic regions are shown in gray, and the BiP‐binding sequence is shown by a horizontal line. (C) Structures of the constant regions of IgG1 and IgE predicted by AlphaFold2 and an enlarged view of the area surrounding amino acids 42–65. (D) Co‐immunoprecipitation of BiP with the chimeric proteins, CD8α‐EEQFN‐GFP and KHHHN‐GFP, in 293T cells. The proportion of the precipitated BiP protein levels normalized with the GFP protein level is shown. Data represent the means with individual data (*n* = 6), *p < 0.05 (Wilcoxon matched‐pairs single‐rank two‐tailed test).

In summary, biochemical analysis, computer‐assisted structural modeling, and BiP‐binding prediction from the primary amino acid sequence suggest that the IgE‐Cε3 region is more likely to bind to BiP and induce ER stress signals compared with the domains of IgG1, and may contribute to isotype‐specific plasma cell differentiation.

## Conflicts of Interest

The authors declare no conflicts of interest.

### Peer Review

The peer review history for this article is available at https://publons.com/publon/10.1002/eji.202451428.

## Supporting information



Supporting Information

## Data Availability

All data supporting the findings of this study are available within the article and/or supplementary information.
