# Supplementary material for: Differential Induction of Endoplasmic Reticulum Stress Signaling by Antibody Isotypes: Implications for Plasma Cell Differentiation
Source: Eur J Immunol. 2025 Apr 25;55(4):e202451428. doi: 10.1002/eji.202451428 (PMC12032515; doi:10.1002/eji.202451428)
Supplement: Supplementary file 1 — Supporting Information [file EJI-55-e202451428-s001.docx]

SUPPORTING INFORMATIONS

Materials and methods

Mice

All experimental protocols were approved by the Animal Care and Use Committee of the University of Occupational and Environmental Health (Approval Number: AE21-007). C57BL/6N mice were obtained from Japan SLC, Inc. and housed in the animal facility at the University of Occupational and Environmental Health under pathogen-free conditions. The mice were handled in compliance with University's guidelines. We used 6–8 week-old male mice for all of the experiments.

Mouse Splenocytes and B Cells Isolation and Culture In Vitro

Mouse splenocytes were isolated as described previously [1]. Mouse B cells were separated from splenocytes using Dynabeads™ Mouse CD43 (Untouched™ B Cells, Invitrogen). Mouse splenocytes or purified B cells were cultured with 5 μg/ml Ultra-LEAF™ purified anti-mouse CD40 antibody (1C10, BioLegend), 10 μg/ml lipopolysaccharides from Escherichia coli O55 (LPS, L4524, Sigma), and 25 ng/ml murine IL-4 (PeproTech) for 4 days in RPMI medium (Wako) containing 10% fetal bovine serum (FBS, Nichirei), 1% penicillin/streptomycin (Wako), and 2 mM L-glutamine (Wako), 1% MEM non-essential amino acids solution (Wako), 1% sodium pyruvate (Wako), and 50 μM 2-mercaptoethanol (Wako). On day 3, the cells were treated with drugs affecting the UPR, including STF-083010 (IRE1α inhibitor, Selleckchem) or GSK2656157 (PERK inhibitor, Selleckchem). Human 293T cells were cultured in DMEM high-glucose medium containing 10% FBS, 1% penicillin/streptomycin, and 2 mM L-glutamine.

Flow Cytometry and Cell Sorting

Single-cell suspensions from in vitro cultures were suspended in PBS containing 2% FBS and stained with the following surface molecule antibodies: CD19-APC (1D3, Tonbo), CD138-APC (281-1, BioLegend), IgE-FITC (23G2, Invitrogen), IgE-PE (RME-1, BioLegend), IgG1-FITC (RMG1-1, BioLegend), TACI-PE (8F10, BioLegend), and CD98-PE (RL388, BioLegend). The stained samples were analyzed using a BD FACSCalibur (BD Biosciences) and sorted using a BD FACSMelody cell sorter (BD Biosciences).

Lentiviral structures and transduction

For lentiviral plasmid construction, PCR-amplified fragments of immunoglobulin were cloned into the pLenti-CMV-MCS-EGFP vector using the In-Fusion® HD Cloning Kit (Promega). For transfection, 2 × 10^5 293T cells were plated overnight in a 24-well plate. The cells were transfected with plasmids using the FuGene® HD Transfection Reagent (Promega). The day after transfection, the cells were analyzed as described in the figure legends.

Immunoblotting and Immunoprecipitation

Cells were collected from the 24-well plate, washed twice in PBS, and cell pellets were lysed using NP-40 lysis buffer with a protease inhibitor cocktail. The cell solution was centrifuged at 15,000 rpm for 10 min at 4°C and the supernatants were collected. For immunoprecipitation, the supernatants were incubated with Dynabeads M-280 sheep anti-mouse IgG coupled with anti-mouse Flag antibody (Invitrogen) for 30 min at 4°C. The immunoprecipitated proteins were separated using DynaMag™-2 (Invitrogen). Thr protein concentration was measured using the Quick Start™ Bradford Protein Assay (Bio-Rad). Approximately 20 µg of protein was separated by 12% or 5%-20% SDS-PAGE and transferred to PVDF membranes (Millipore) at 25 V for 1 h. After blocking with 5% skim milk, the primary antibodies were incubated at 4°C overnight in Can Get Signal solution 1 (TOYOBO). Secondary antibodies were incubated for 1 h at room temperature in Can Get Signal solution 2 (TOYOBO). The primary antibodies included anti-human BiP (Cell Signaling Technology) at a 1:2000 dilution, anti-GFP (MBL) at a 1:2500 dilution, and Direct-Blot™ anti-β-Actin HRP (BioLegend) at a 1:100000 dilution. The secondary antibody was HRP-conjugated anti-rabbit IgG at a 1:5000 dilution. The blots were imaged using ImageQuant LAS 4000 (Cytiva) with Amersham ECL Select (Cytiva) and the data were analyzed using ImageJ software.

Quantitative Real-Time PCR

Total RNA was isolated from the lysed cells using Sepasol-RNA I Super G (Nacalai Tesque) based on the manufacturer's instructions. cDNA was synthesized from RNA using the ReverTra Ace® qPCR RT Master Mix with gDNA Remover (TOYOBO) based on the manufacturer's instructions. Quantitative RT-PCR was performed on cDNA using the THUNDERBIRD SYBR® qPCR Mix (TOYOBO) and the Thermal Cycler Dice Real Time System Lite (Takara). The relative levels of the target mRNAs were normalized to β-actin and calculated using the $2^{-\Delta\Delta Ct}$ method. All primers were provided by FASMAC. The primer sequences were as follows:

Target		Forward	Reverse
Human	XBP1 unspliced	5′-TGCTGAGTCCGCAGCACTCA-3′	5′-GCTGGCAGGCTCTGGGGAAG-3′
	XBP1 spliced	5′-TGCTGAGTCCGCAGCAGGTG-3′	5′-GCTGGCAGGCTCTGGGGAAG-3′
	BiP	5′-TCTTGTTGGTGGCTCGACTC-3′	5′-ATCTGGGTTTATGCCACGGG-3′
	ACTB	5′- ATTGGCAATGAGCGGTTC-3′	5′- CGTGGATGCACAGGACT-3′.
Mouse	XBP1 unspliced	5′-TGCTGAGTCCGCAGCACTCA-3′	5′-ACTAGCAGACTCTGGGGAAG-3′
	XBP1 spliced	5′-TGCTGAGTCCGCAGCAGGTG-3′	5′-ACTAGCAGACTCTGGGGAAG-3′
	CHOP	5′-CCAGAATAACAGCCGGAACCT-3′	5′-CTGCTTTCAGGTGTGGTGGT-3′
	ACTB	5′-CATCCGTAAAGACCTCTAGCCAAC-3′	5′-ATGGAGCCACCGATCCACA-3′

Hydrophobicity Analysis

The hydrophobicity of each amino acid was analyzed using the Kyte-Doolittle scale index for computational hydrophobicity analysis.

Hsp70 Chaperone Binding Prediction

All BiP-binding sequences in the protein were identified by ChaperISM, which uses a position-independent scoring matrix derived from previously published data.

Three-Dimensional Structure Prediction

All proteins were analyzed based on their amino acid sequences using AlphaFold2, which is a computational method that can predict protein structure with atomic accuracy.

Statistical Analysis

All statistical analyses were performed using GraphPad Prism 8. For a comparison of two groups, the Mann-Whitney two-tailed test, paired two-tailed t-test, unpaired two-tailed t-test, and Wilcoxon matched-pairs signed rank two-tailed test were used. Data are presented as the mean ± SEM. P-values less than 0.05 were considered statistically significant.

Author contributions

K.O. designed the study, performed and analyzed all experiments, and wrote the paper. K.S. performed experiments. T.D. and M.E. designed and supervised the study and wrote the paper.

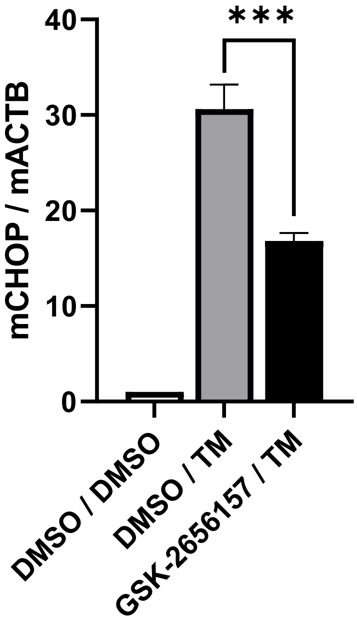

Figure S1: Effect of GSK-2656157 on CHOP mRNA induction in B cells.
Splenic B cells were cultured with anti-CD40 antibody and IL-4 for 3 days. GSK-2656157 or DMSO was added prior 30 min. before tunicamycin (TM) treatment. The cells were harvested 4 hours after TM addition. Relative ratios of mCHOP mRNA to mACTB mRNA are graphed. Data represent means ± SD from three experiments. ****P* < 0.001(Student t-test).

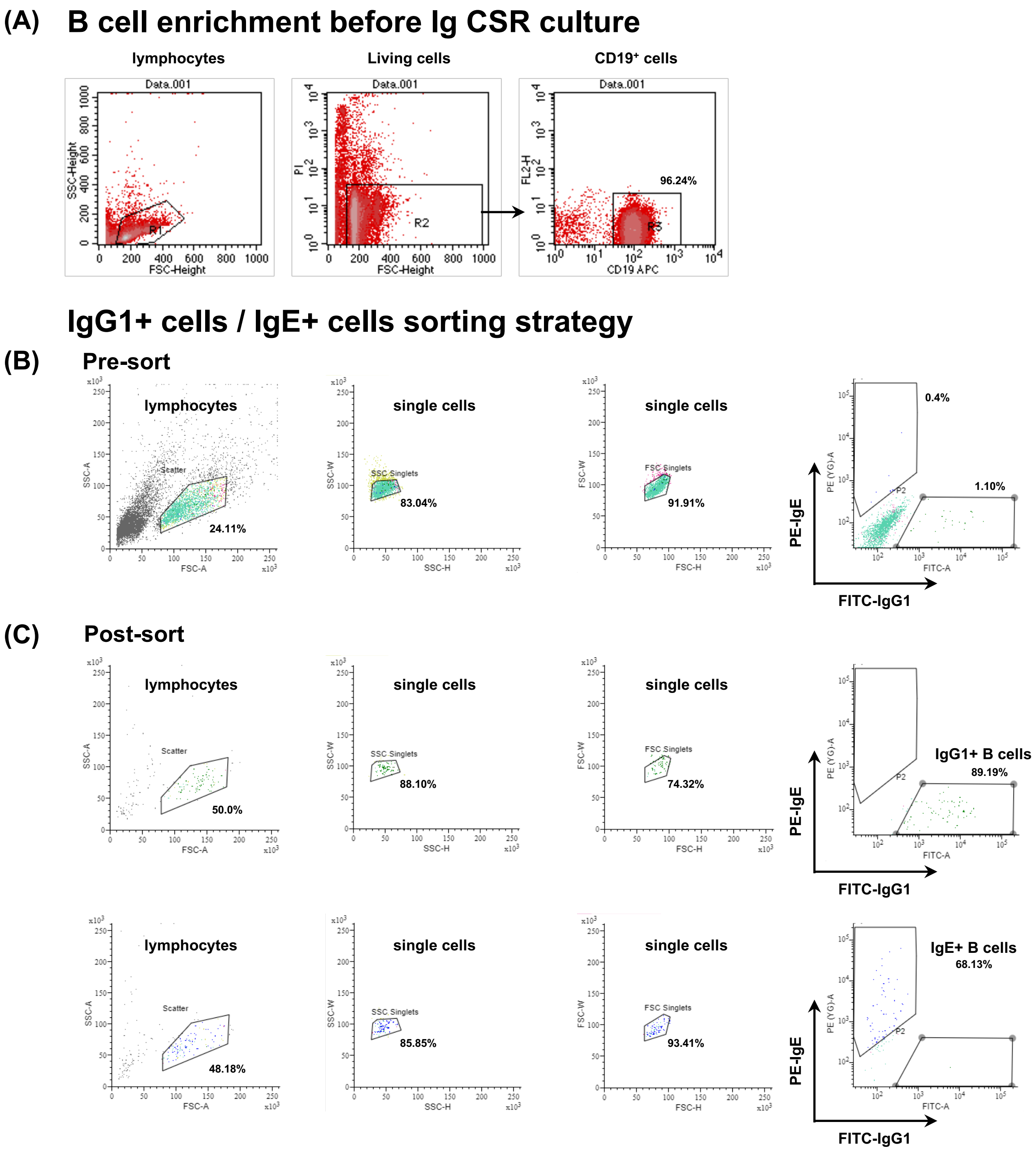

Figure S2: Sorting strategy and purity of enriched B cells. (A) FACS profiles of purified B cells separated from splenocytes with MACS magnetic beads. (B) FACS profiles of pre-sorted B cells cultured with anti-CD40 antibody and IL-4 for 4 days. (C) FACS profiles of post-sorted IgG1+ and IgE+ B cells.

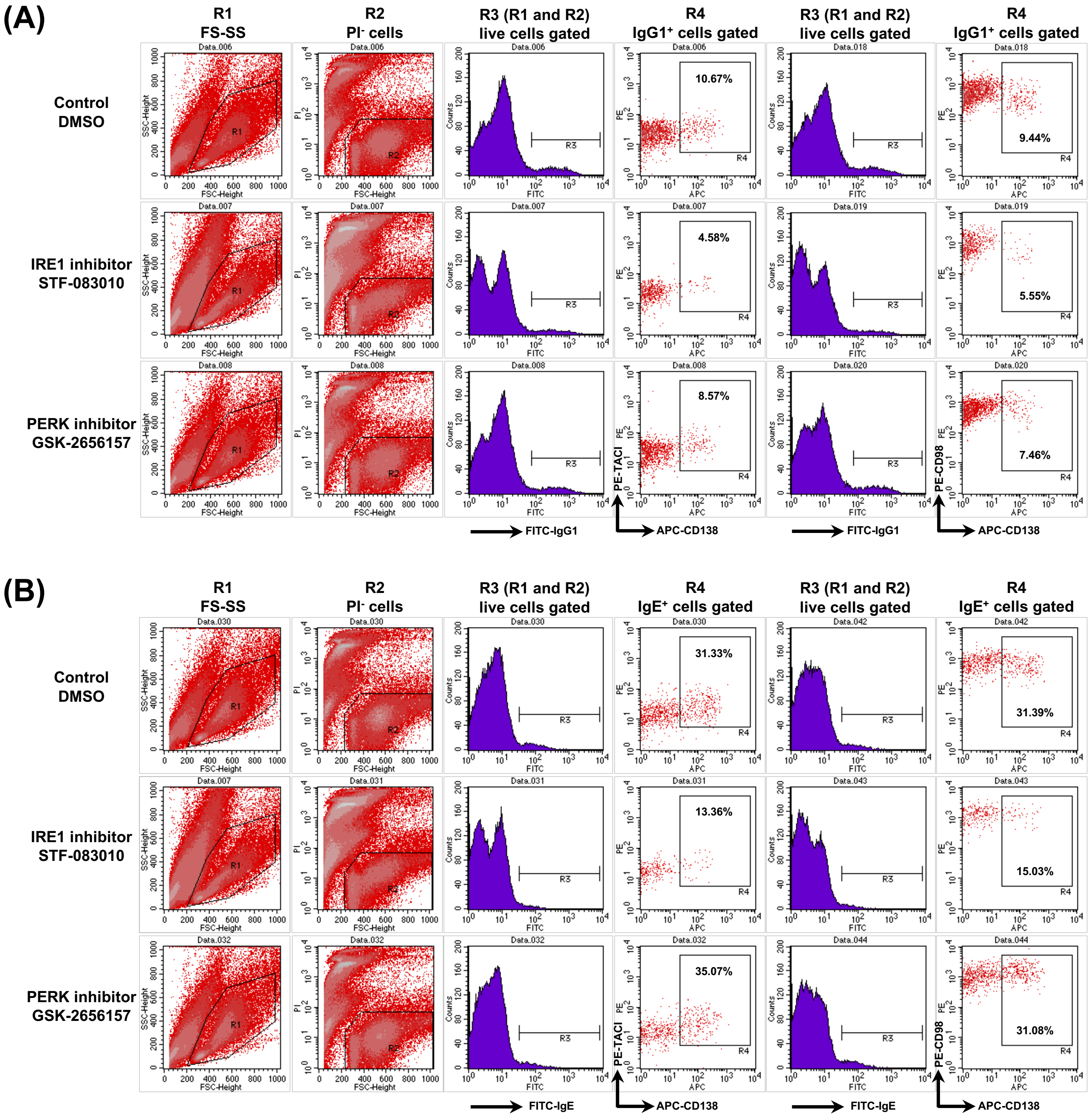

Figure S3: Plasma cells differentiation is dependent on IRE1 signaling.
Splenic B cells were cultured for 4days with anit-CD40 antibody and IL-4 with DMSO, STF-083010 or GSK-2656157. Cells were stained anti-IgG1, IgE, CD138, TACI, CD98, and PI. FACS profiles of CD138/TACI or CD138/CD98 in IgG1+ cells (A) or in IgE+ cells (B) are shown.

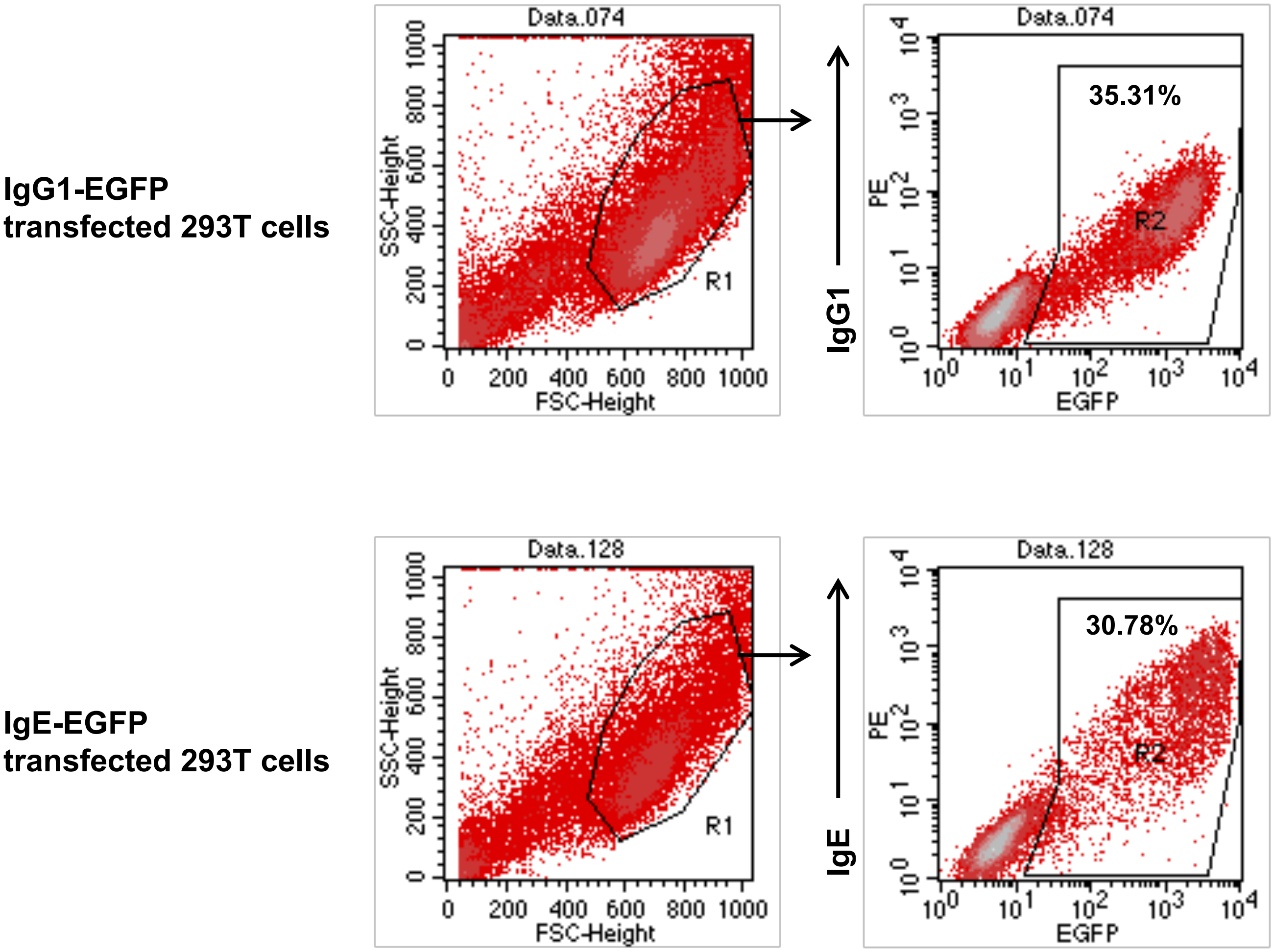

Figure S4: Transfection efficiency of CD8α-IgG1-EGFP and CD8α-IgE-EGFP.
293T cells transfected with CD8α-IgG1-EGFP and CD8α-IgE-EGFP expressing plasmid. The cells were stained with anti-IgG1 or IgE antibody and analyzed with FACS.

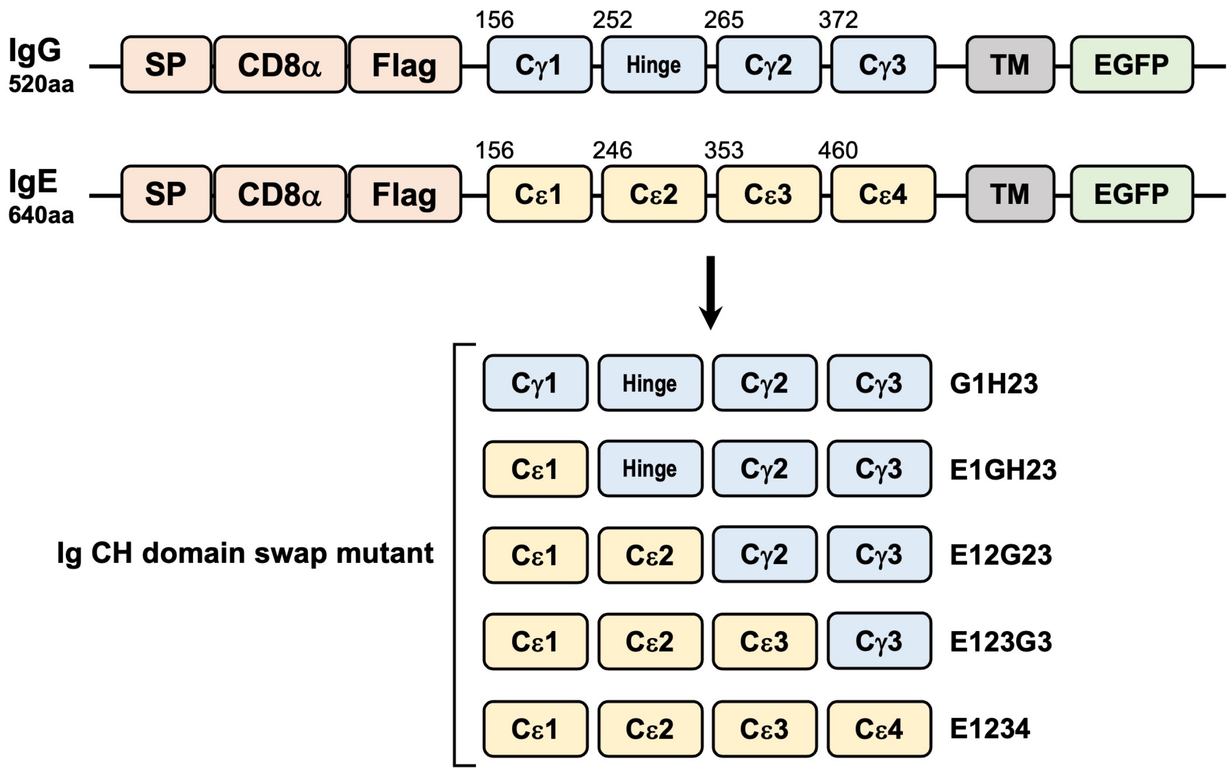

Figure S5

Schematic diagram of chimeric immunoglobulin constant region constructs. SP; signal sequence of CD8α, TM; transmembrane. Numbers indicated above each construct are number of amino acid residue of mIgG1 (GenBank accession number BBE36239.1) and mIgE (AAB06744.1). Domain swapping constructs was made as shown below.

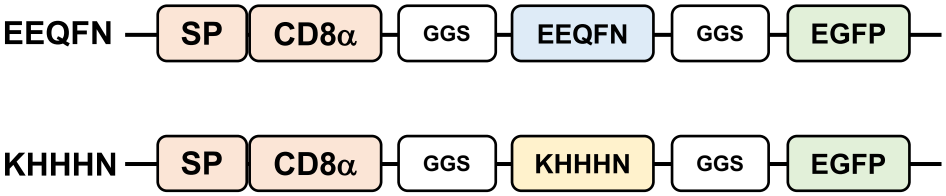

Figure S6

Schematic diagram of chimeric proteins containing only the motif of IgG1 (EEQFN) and IgE (KHHHN) constructs. SP; signal sequence of CD8α, GGS; elongated linker.

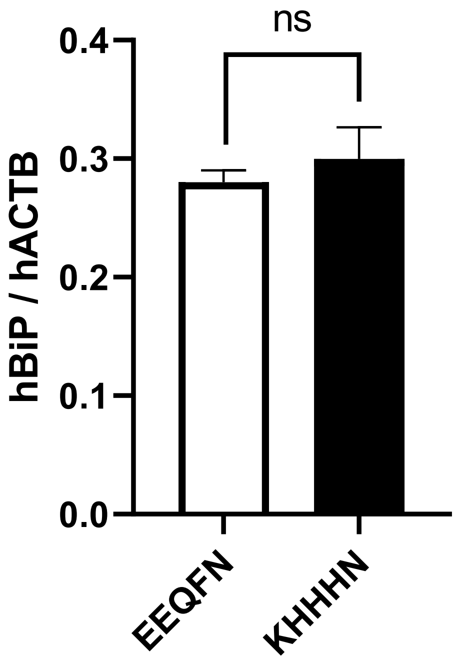

Figure S7 BiP mRNA induction in 293T cells.
Plasmids expressing CD8α-EEQFN-GFP, CD8α-KHHHN-GFP were introduced into 293T cells. After 24 hours, BiP mRNA was quantified by real-time PCR. The BiP mRNA levels normalized with ACTB mRNA levels are graphed. Data presented means ± SD (n=3).

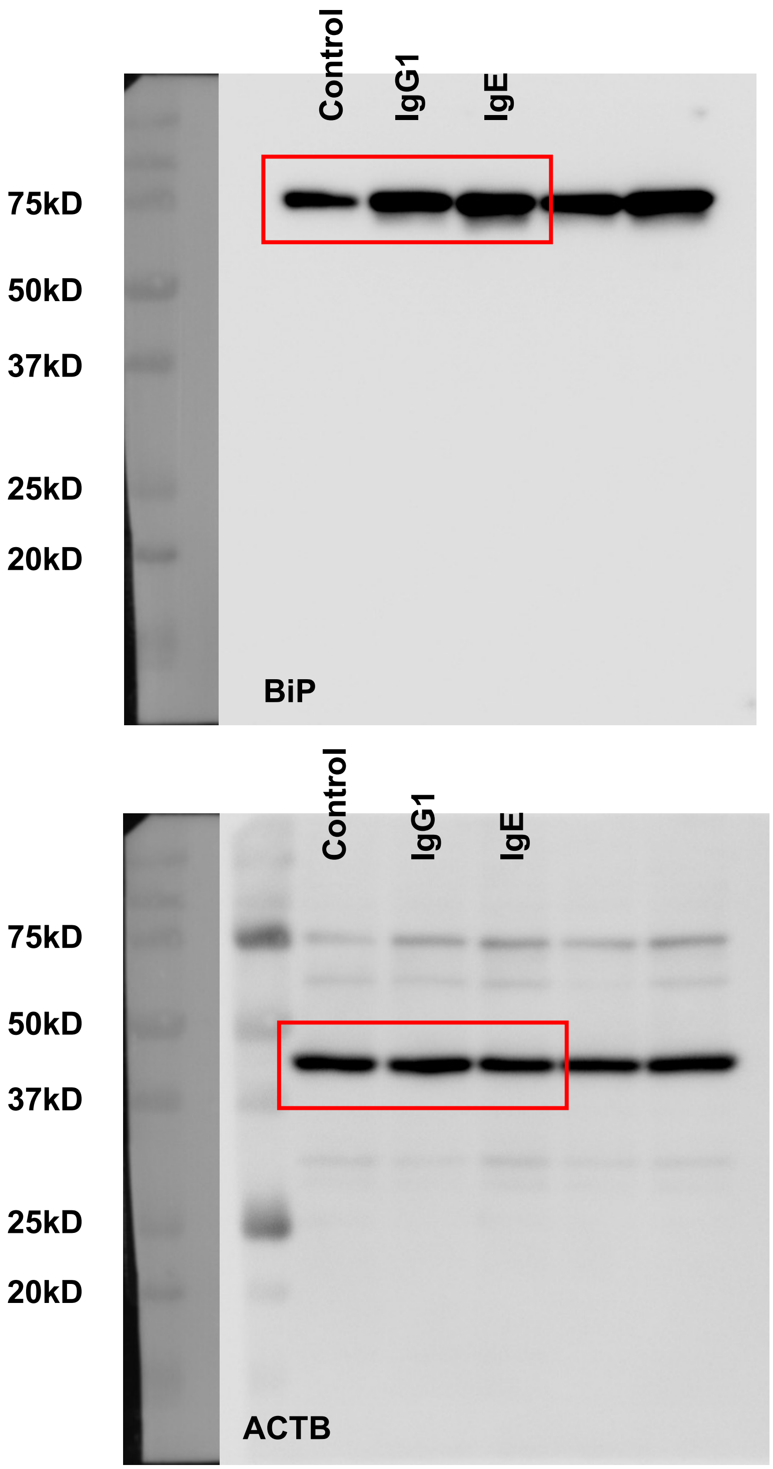

Figure S8
Full gel images corresponding to Figure 1C.

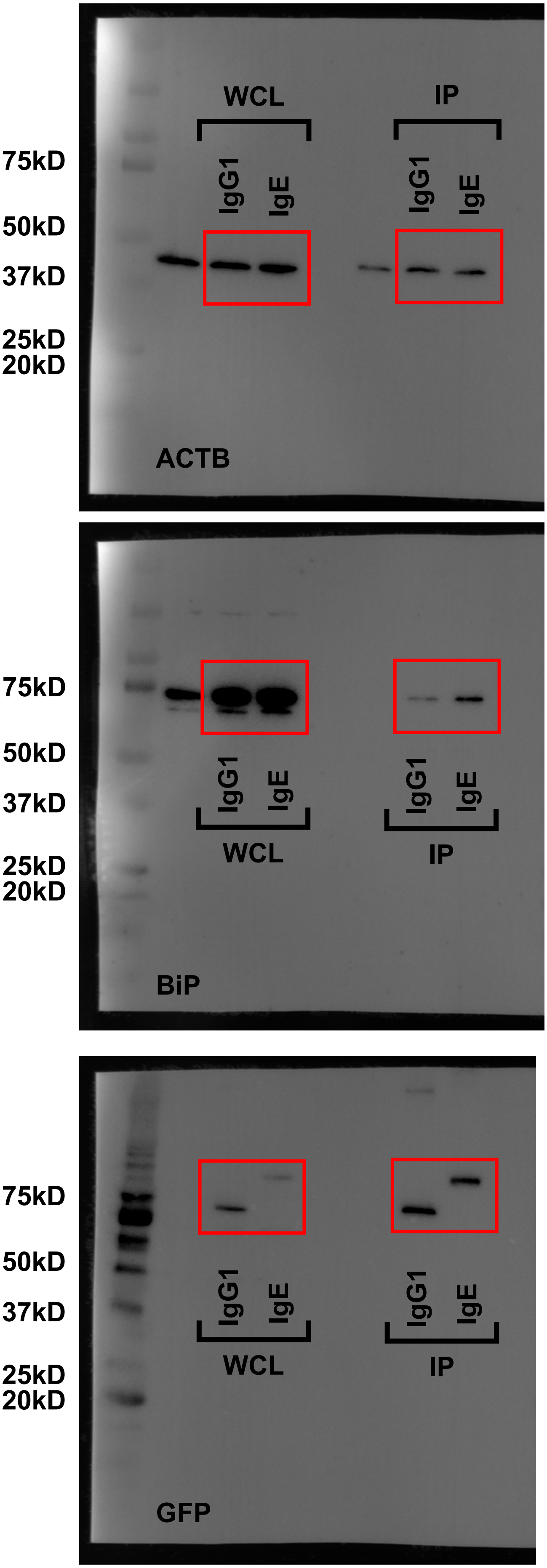

Figure S9
Full gel images corresponding to Figure 1D.

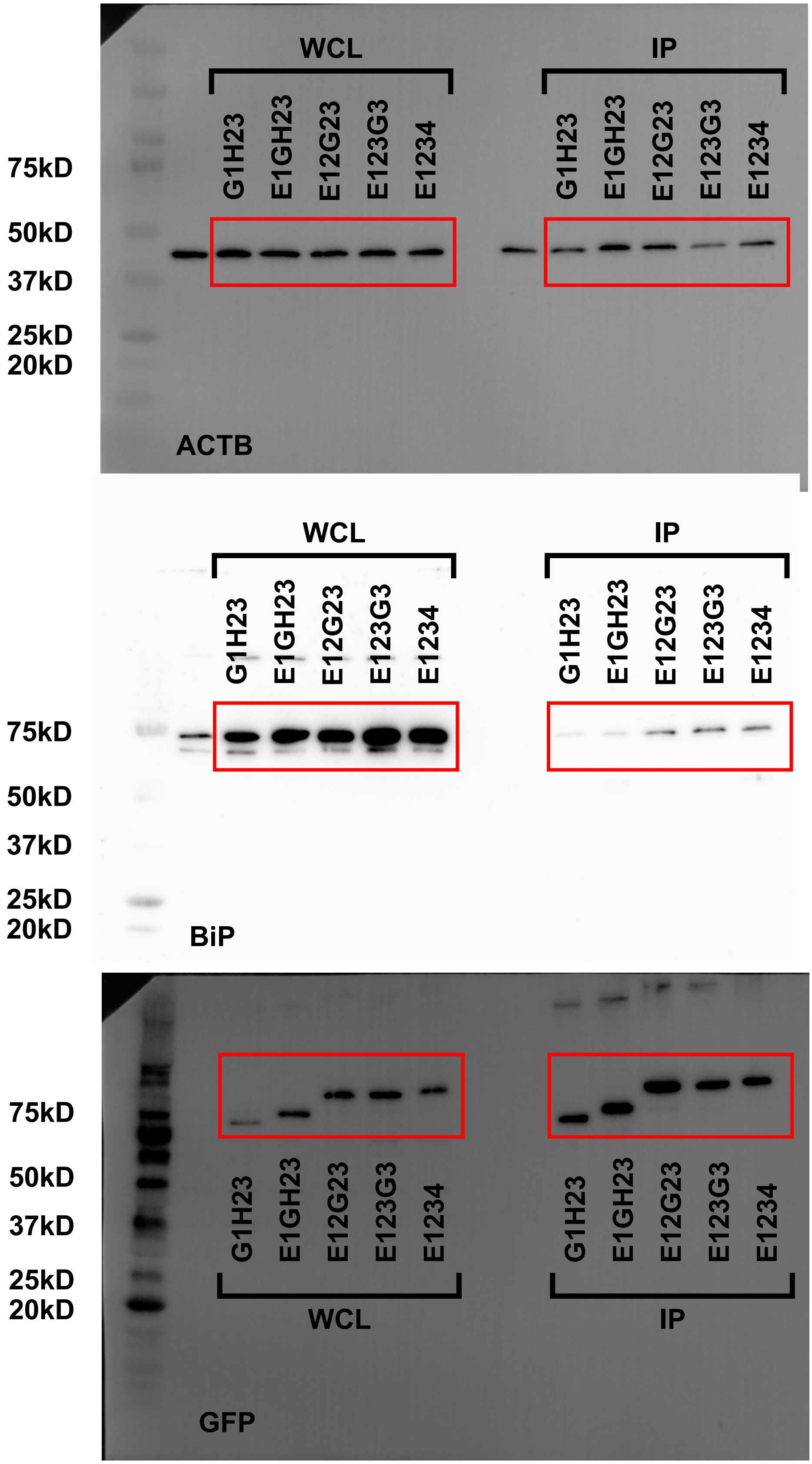

Figure S10
Full gel images corresponding to Figure 1F.

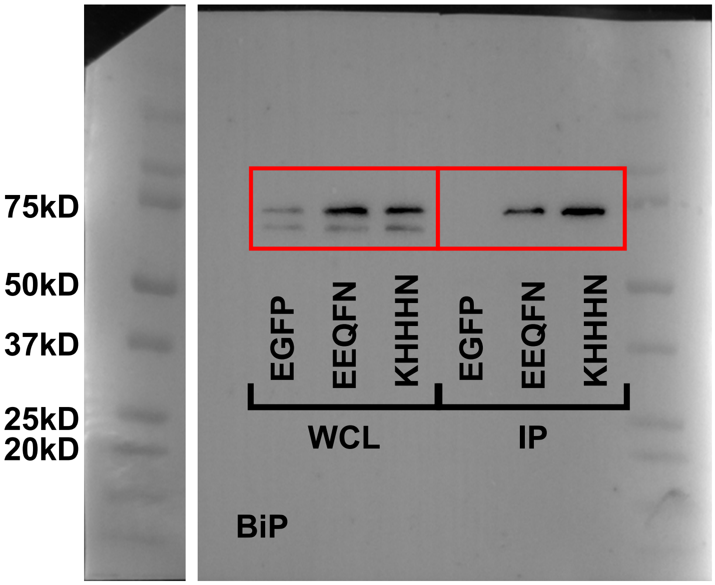

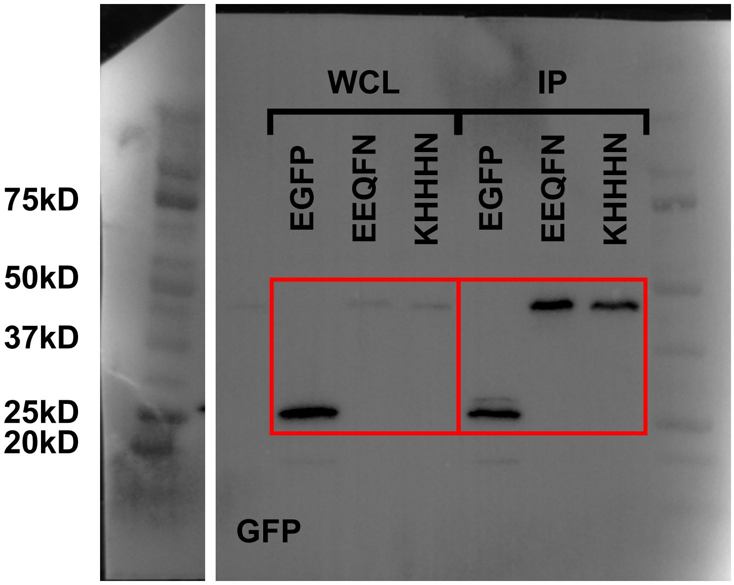

Figure S11
Full gel images corresponding to Figure 2D.
